# Exploring transparent reporting and data availability in systematic reviews to identify subgroup evidence: imaging for suspected hepatocellular carcinoma in the non-cirrhotic liver

**DOI:** 10.1186/s13023-024-03356-x

**Published:** 2024-10-02

**Authors:** Michiel S. Oerbekke, Robert A. de Man, Frederike G. I. van Vilsteren, Maarten W. Nijkamp, Eric Tjwa, Charlotte M. W. Gaasterland, Maarten J. van der Laan, Lotty Hooft

**Affiliations:** 1 Knowlegde Institute of the Dutch Association of Medical Specialists, Utrecht, The Netherlands; 2 Cochrane Netherlands, Julius Center for Health Sciences and Primary Care, University Medical Center Utrecht, Utrecht University, Utrecht, The Netherlands; 3 Department of Gastroenterology and Hepatology, Erasmus Medical Center, Rotterdam, The Netherlands; 4 Department of Gastroenterology and Hepatology, University Medical Center Groningen, Groningen, The Netherlands; 5 Department of Surgery, University Medical Center Groningen, Groningen, The Netherlands; 6 Department of Gastroenterology and Hepatology, Radboud University Medical Centre, Nijmegen, The Netherlands; 7 Julius Center for Health Sciences and Primary Care, University Medical Center Utrecht, Utrecht University, Utrecht, The Netherlands

**Keywords:** Systematic reviews, Clinical practice guidelines, Rare diseases, Subgroups, Reporting guidelines, Data availability, Data accessibility, Data reusability, Resource utilization

## Abstract

We aim to illustrate the role of complete and transparent reporting coupled with access to data sourced from published systematic reviews, especially assisting in the identification of evidence for subgroups within the context of a rare disease. To accomplish this principle, we provide a real-world example encountered during the revision of the Dutch clinical practice guideline for hepatocellular carcinoma. Specifically, we retrieved insights from two Cochrane reviews to identify direct evidence concerning the diagnostic test accuracy of computed tomography and magnetic resonance imaging for detecting hepatocellular carcinomas in suspected patients without liver cirrhosis. Through reusing the Cochrane review authors’ efforts already undertaken in their exhaustive literature search and selection, we successfully identified relevant direct evidence for this subgroup of suspected patients without cirrhosis and performed an evidence synthesis within the constraints of limited resources for the guideline revision. This approach holds the potential for replication in other subgroups in the context of rare diseases, contingent on the transparent and complete reporting of systematic reviews, as well as the availability and accessibility of their extracted data. Consequently, we underscore the importance of adhering to established reporting guidelines for systematic reviews, while simultaneously advocating for increased availability and accessibility to data. Such practices would not only increase the transparency and reproducibility of systematic reviews but could also increase reusability of their data. In turn, the increased reusability could result in reduced resource utilization in other sectors such as the guideline developing community as we show in our example.

To the editor

In 2022 the incidence of patients with hepatocellular carcinoma (HCC) in the Netherlands was n = 649 (502 males, 147 females) with a five-year prevalence of n = 1303, based on preliminary data in the Netherlands Cancer Registry [1]. This qualifies as a rare disease, given that the population size in the Netherlands was N = 17.815.508 as of December 2022 [2]. Liver cirrhosis is usually observed in patients with HCC, although the disease may also develop without cirrhosis in an even smaller group of patients. This may vary across populations, where 12% [3] and 26% [4] of the patients with HCC did not have liver cirrhosis within studies in the United States and Taiwan, respectively. Diagnostic imaging indicators of HCC in patients without liver cirrhosis have a lower specificity compared to those in patients with cirrhosis [5]. International clinical practice guidelines (CPGs) strongly recommend a pathology-confirmed diagnosis for the subgroup without cirrhosis [5, 6], a practice that appears to be comparatively more prevalent among these patients [3, 4].

Recently, there has been a revision of the Dutch HCC diagnosis and treatment CPG from its prior publication in 2013. The multidisciplinary CPG panel wished to provide guidance regarding the utilization of solely computed tomography (CT) or magnetic resonance imaging (MRI) for diagnosing HCCs in suspected patients without liver cirrhosis, as an alternative to a pathology-based diagnosis. We developed this guidance as a dedicated segment within the CPG, supported by a comprehensive synthesis of the evidence. However, a literature specialist demonstrated that even a specific search strategy for our evidence synthesis would result in a greater number of primary studies (i.e. n = 1813 from Embase and n = 1383 from Ovid/MEDLINE, duplicates not removed) than our capacity allowed for processing within a single CPG segment when considering the available resources for the CPG revision. Consequently, an alternative search strategy was developed which centered around published systematic reviews (SRs), resulting in n = 33 hits after removing duplicates. The search strings for Embase and Ovid/MEDLINE involved keywords for HCC, CT, MRI, histology, diagnostic accuracy, and SRs and is available upon request via www.richtlijnendatabase.nl. One of the involved CPG panel members selected 10 potentially relevant SRs based on their titles and abstracts (TIAB), including two Cochrane Diagnostic Test Accuracy reviews [7, 8]. The supporting CPG methodologist read these 10 SRs in full-text and determined with the involved CPG panel members that none of these SRs reported relevant and direct information for the subsample of patients without cirrhosis. Therefore, none of the SRs could be directly used as an evidence synthesis in the CPG segment for the Dutch HCC CPG.

Both Cochrane reviews, however, reported the number of included studies that had 100% prevalence of cirrhosis and conducted a sensitivity analysis for studies with a prevalence of > 90% versus studies < 90% prevalence [7, 8]. This indicated that the Cochrane reviews captured primary studies that also recruited suspected patients without liver cirrhosis. These studies could possibly report (sub) analyses for the group of patients without cirrhosis. The Cochrane reviews transparently reported their search strategy, including sensitive search strings. The CPG methodologist judged that these search strategies (see Table 1) indeed could encase the retrieval of studies with a (sub)sample of patients without liver cirrhosis.

**Table 1:** Summary of the search and retrieval of the two Cochrane diagnostic test accuracy reviews.

Author, year	Modality	Generalized search string*	Search retrieval after removing duplicates (n)	TIAB potentially relevant (n)	TIAB excluded (n)	Full-text included (n)	Full-text excluded (n)
Nadarevic [8]	CT	(CT) AND (HCC) AND (liver disease)	25,230	165	25,065	21	144
Nadarevic [7]	MRI	(MRI) AND (HCC) AND (liver disease OR cirrhosis)	9661	117	9544	34	83

*This is a generalized version of the search sting without the synonyms the authors have used. There are differences within each search string depending on which database was sought. The complete search strings are available in the original publication of the Cochrane Diagnostic Test Accuracy reviews

*CT* Computed Tomography, *HCC* Hepatocellular Carcinoma, *MRI* Magnetic Resonance Imaging, *n* number of studies, *TIAB* Title/abstract

We reused the potentially relevant hits from both Cochrane reviews, comprising of the included studies and the referenced excluded studies. For the included studies, we downloaded the Cochrane reviews’ data files from the Cochrane Library. These files contained data about the prevalence of cirrhosis in the included studies. We selected those studies which had a prevalence of < 100% or when the prevalence was not reported (n = 12), since these could potentially report (sub)analyses for patients without cirrhosis. The CPG methodologist read these papers full text and determined with the involved GPC panel members that two studies [9, 10] met the inclusion criteria for our evidence synthesis. Given that the full-text excluded studies from the Cochrane reviews were explicitly cited, we were able to compile and access this set of n = 214 studies (duplicates removed). One CPG panel member screened these primary studies on TIAB and identified a set of n = 18 potentially relevant studies for our evidence synthesis. The CPG methodologist read these 18 studies full-text and determined with the involved CPG panel members that one additional study [11] met the inclusion criteria. Overall, three studies met the inclusion criteria of our evidence synthesis for uptake in the GCP segment for the Dutch HCC CPG revision. The process is visualized in Fig. 1.

**Fig. 1:**
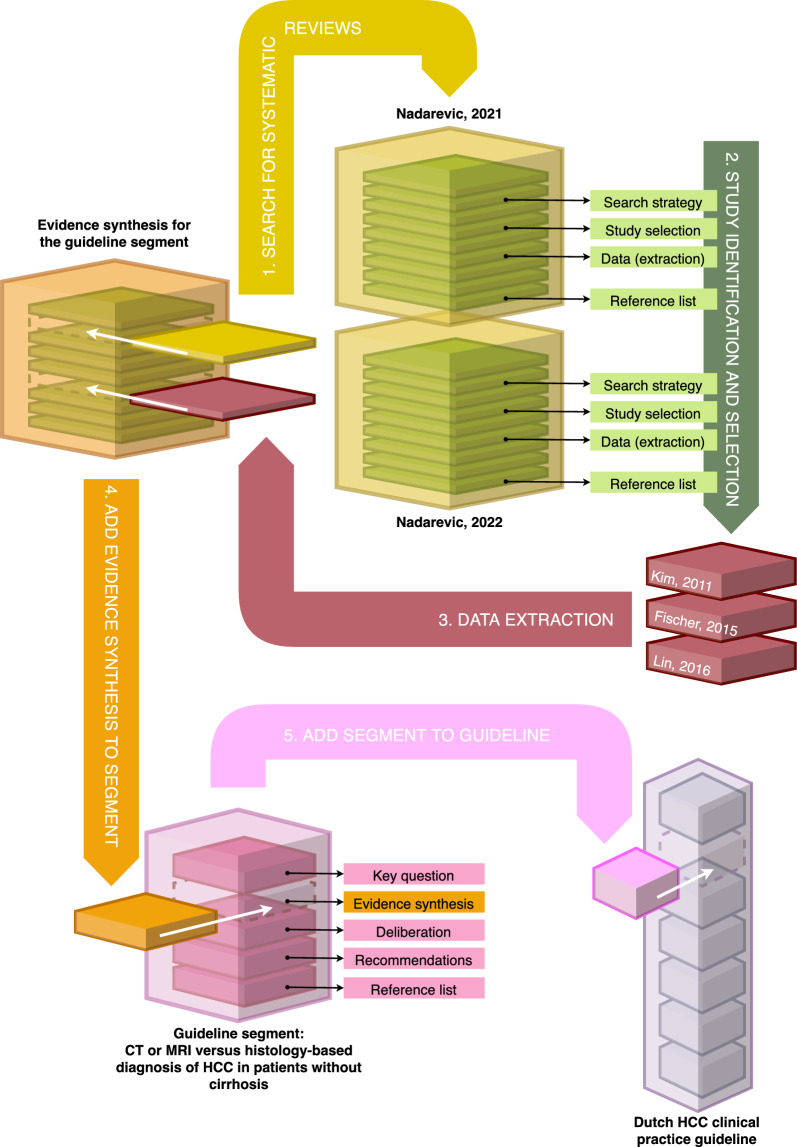
Visualization of the process leading to uptake of the studies in the Dutch HCC guideline*.* We searched for published systematic reviews in our evidence synthesis for the Dutch hepatocellular carcinoma (HCC) clinical practice guideline segment (step 1). This resulted in the identification of two Cochrane diagnostic test accuracy reviews that also seemed to capture studies with a (sub)sample of suspected patients without liver cirrhosis. Through the transparently reported information and available data in these Cochrane reviews, we were able to identify three studies with direct evidence regarding the diagnostic test accuracy of computed tomography (CT) or magnetic resonance (MRI) in patients suspected of an HCC with a non-cirrhotic liver (step 2). Relevant data was extracted from these three studies for our own evidence synthesis (step 3). The evidence synthesis was then added to the guideline section for its development (step 4). Once endorsed, the guideline segment is added to the Dutch HCC clinical practice guideline (step 5) comprising of segments covering various other topics in the diagnosis and treatment of HCC (available via www.richtlijnendatabase.nl)

Through this method, we effectively have reused the Cochrane review authors’ efforts in the literature search, the literature screening, and the data-extraction to identify direct evidence for the test accuracy of CT and MRI in the subgroup of patients without liver cirrhosis. This was only possible because of the transparent reporting of information by the Cochrane review authors and the availability and accessibility of data within both reviews. It is important to note that this method does not extend to identifying primary studies published after the search dates reported in the Cochrane reviews. Nevertheless, it provided a systematic and time-efficient strategy for searching and screening potentially relevant literature up to the Cochrane reviews’ search dates. The TIABs of 33 systematic reviews and 214 primary studies were screened instead of n = 25,230 [8] and n = 9661 [7] primary studies for CT and MRI, respectively. With an estimate that manually screening 60 to 120 TIABs takes approximately an hour [12], screening the Cochrane reviews’ search retrieval would take about 210–420 h for CT and 80–161 h for MRI (not regarding potential duplicates). Our own initial search strategy for primary studies would have taken about 15–30 h to screen on TIAB for the retrieval from Embase alone. However, through reusing the Cochrane authors’ efforts, the involved CPG panel members invested far less time in screening the TIABs for our evidence synthesis in the CPG revision, which would be about 2–4 h according to the estimate of 60 to 120 abstract per hour. The CPG segment (including its supporting evidence synthesis with data extraction table and exclusion table) was published online at www.richtlijndatabase.nl.

The European Association for the Study of the Liver [5] and European Society for Medical Oncology [13] CPGs for HCC from 2018 do not seem to reference any of these primary studies for the test accuracy of CT or MRI in patients without cirrhosis. The latest American Association for the Study of Liver Diseases CPG from 2023 [6], however, does reference one of the identified primary studies [9]. The American CPG referenced an additional primary study [14] which we had not identified through both Cochrane reviews. This particular study was published after the search date in one of the Cochrane reviews [8], making it impossible to be included. However, it was published shortly before the last search date in the second Cochrane review [7]. The study might not yet have been indexed properly in the databases this short before the last search date in the Cochrane review, resulting in its omission. Alternatively, in both Cochrane reviews, the authors exclusively included studies that reported per-patient analyses because they were interested in the ability of CT and MRI to detect patients with HCC, while per-lesion analyses typically provides information about staging [7, 8]. The study might have been excluded during the TIAB screening phase based on its abstract, which suggested that a per-lesion analysis was used. The reported Ovid MEDLINE search string [7] is, however, capable of capturing the study as it was found when we reproduced the search in Ovid MEDLINE on the 24th of July 2023. In this situation, having access to the complete search retrieval would have been beneficial allowing us to check whether the study was absent in the retrieval altogether or was excluded in the TIAB screening phase.

With the hereby described process during the Dutch HCC CPG revision, we have shown how information and data in SRs was used by the CPG working group and methodologist to identify direct evidence for a subgroup in a rare disease while under resource constraints. The potential for such a process to be applicable to subgroups in other rare diseases depends on the transparent and complete reporting of information in SRs, alongside with the availability of extracted data. This is essential for two reasons. Firstly, it allows for the verification whether the search strategy could have captured the subgroup of interest, and to understand which and when databases were last searched. Secondly, having access to this information and data are necessary to identify and retrieve the relevant literature for your own evidence synthesis. We therefore stress the importance of adhering to reporting guidelines for SRs, such as the PRISMA statement [15] and its extensions. Moreover, we emphasize the value of providing extracted data from primary studies in a data file format that can be accessed using widely available or free software. Although authors generally acknowledge the importance of the PRISMA statement’s items [16], existing evidence indicates that adherence to these reporting guidelines is suboptimal [17, 18]. However, enhanced adherence does not only increase transparency and reproducibility of SRs but may, combined with sufficient data availability and accessibility, also increase the reusability of data within SRs. The integration of efforts between primary research, evidence synthesis, and clinical practice guideline development is crucial. Currently, these processes and efforts seem to be performed siloed [19]. In our approach, by being able to reuse efforts in the production of SRs and the data within these SRs, we have successfully bridged a notable gap between evidence synthesis and CPG development while optimizing resource utilization during the production of a CPG.

## Data Availability

The clinical practice guideline segment (in Dutch), including its evidence synthesis (in English), is available at www.richtlijnendatabase.nl. The search string used in the search strategy is available upon request via www.richtlijnendatabase.nl. The Cochrane diagnostic test accuracy reviews and their statistical data files are stored in the Cochrane Database of Systematic Reviews (CDSR, ISSN 1469-493X) and can be found through the Cochrane Library at www.cochranelibrary.com.

## Abbreviations

CPG: Clinical practice guideline
CT: Computed tomography
HCC: Hepatocellular carcinoma
MRI: Magnetic resonance imaging
SR: Systematic review
TIAB: Title/abstract
